# Falls in Community-Dwelling Older Adults with Lower Back or Knee Pain Are Associated with Cognitive and Emotional Factors

**DOI:** 10.3390/ijerph17144960

**Published:** 2020-07-09

**Authors:** Tatsuya Hirase, Hyuma Makizako, Yoshiro Okubo, Stephen R. Lord, Minoru Okita, Yuki Nakai, Toshihiro Takenaka, Takuro Kubozono, Mitsuru Ohishi

**Affiliations:** 1 Department of Physical Therapy Sciences, Nagasaki University Graduate School of Biomedical Sciences, Nagasaki 852-8520, Japan; htatsuya@nagasaki-u.ac.jp (T.H.); mokita@nagasaki-u.ac.jp (M.O.); 2 Department of Physical Therapy, School of Health Sciences, Faculty of Medicine, Kagoshima University, Kagoshima 890-8544, Japan; nakai@health.nop.kagoshima-u.ac.jp; 3 Falls, Balance and Injury Research Centre, Neuroscience Research Australia, Sydney, NSW 2031, Australia; y.okubo@neura.edu.au (Y.O.); s.lord@neura.edu.au (S.R.L.); 4 Tarumizu Municipal Medical Center, Tarumizu Chuo Hospital, Kagoshima 891-2124, Japan; takenaka@tarumizumh.jp; 5 Department of Cardiovascular Medicine and Hypertension, Graduate School of Medical and Dental Science, Kagoshima University, Kagoshima 890-8520, Japan; kubozono@m.kufm.kagoshima-u.ac.jp (T.K.); ohishi@m2.kufm.kagoshima-u.ac.jp (M.O.)

**Keywords:** aged, accidental falls, pain, mild cognitive impairment, depressive symptoms

## Abstract

(1) Background: The present study aimed to examine physical, cognitive and emotional factors affecting falls in community-dwelling older adults with and without pain; (2) Methods: Data from 789 older adults who participated in a community-based health survey were analyzed. Participants completed questionnaires on the presence of pain and previous falls. Muscle weakness (handgrip strength < 26.0 kg for men and < 18.0 kg for women) and low skeletal muscle mass (appendicular skeletal muscle mass index < 7.0 kg/m^2^ for men and < 5.7 kg/m^2^ for women) were determined. Mild cognitive impairment (MCI) and depressive symptoms were assessed using the National Center for Geriatrics and Gerontology-Functional Assessment Tool and 15-item geriatric depression scale (GDS-15), respectively; (3) Results: In participants with pain, MCI and GDS-15 were associated with previous falls after adjusting for age, sex, education and medication use. In participants without pain, muscle weakness and low skeletal muscle mass were associated with previous falls when adjusting for the above covariates; (4) Conclusions: Falls in participants with pain were associated with cognitive and emotional factors, whereas falls in those without pain were associated with physical factors. Fall prevention interventions for older adults with pain may require tailored strategies to address cognitive and emotional factors.

## 1. Introduction

More than one third of people aged 65 years or older fall at least once per year, with about half doing so recurrently [1]. Between 20% and 30% of those who fall suffer moderate to severe injuries, including fractures and head trauma, which can lead to disability, early admission to nursing homes and even death [2,3].

Pain is also common in community-dwelling older adults, with prevalence rates ranging from 37% to 53% [4,5]. Pain has been identified as a significant risk factor for falls in community-dwelling older adults, and systematic review evidence suggests those with pain have a two-fold increased risk of falling compared to those without pain [6,7]. Among the general older population, reduced physical function, cognitive impairment and depressive mood have also been identified as risk factors for falls [3,8,9]. However, reasons why older adults with pain are at such increased risk of falling are not well understood, therefore limiting our ability to design an effective fall prevention approach for older adults with pain.

The impact of pain on physical function has been relatively well documented [4,10]. Our systematic review evidence has suggested that pain is associated with poor dynamic, static, multicomponent and reactive balance measurements [11], all of which can contribute to an increased fall risk [1]. Furthermore, pain may also influence the risk of falling, through the psychological pathway. It has been reported that pain is associated with poor cognition and impaired executive function, inattention and depressed mood [12,13,14]. Since executive function and depression have been identified as risk factors for falls [8,15], the above findings suggest cognitive and emotional factors may also play a role in increasing fall risk in older people with pain. However, further research is required to identify risk factors for falls in this group and whether such risk factors differ from those found in older people without pain. 

Therefore, the aim of this cross-sectional study was to determine whether risk factors for falls differ between community-dwelling older adults with and without pain while adjusting for relevant covariates. This information may be useful for facilitating fall prevention strategies for both those with and without pain.

## 2. Materials and Methods 

### 2.1. Participants

This study utilized cross-sectional data conducted as part of the Tarumizu Study, a longitudinal study that has been conducted jointly by Kagoshima University (Faculty of Medicine), Tarumizu City Office and Tarumizu Chuo Hospital since 2017 [16]. The Tarumizu Study 2018 was conducted between July and December 2018 as a community-based health survey for older adults living in Tarumizu City, a local city of Kagoshima, Japan. A total of 859 adults aged ≥ 65 years participated in the survey. Data for participants with diagnosed dementia (n = 19), stroke (n = 19) and Parkinson’s disease (n = 3) were excluded. Outcome data were also missing for an additional 29 participants. Thus, data from 789 participants (mean age 74.8 years, 64.1% women) were analyzed. Informed consent was obtained from all participants before study participation, and the ethics committee of the Faculty of Medicine, Kagoshima University approved the study protocol (ref no. 170351).

### 2.2. Assessments

Prior to commencing the study, all staff were trained by a study author regarding the assessment protocol to ensure consistency across the staff members. 

#### 2.2.1. Pain

Pain was assessed with the question “Do you currently have lower back or knee pain?” These pain sites were chosen as they have a high prevalence in older adults, and because they are the main pain sites that lead to mobility disability in this population [4]. Consistent with previous studies [10,17], participants with either lower back or knee pain were defined as the “pain” group, while participants without lower back or knee pain were defined as the “nonpain” group.

#### 2.2.2. Falls

Falls were assessed with the question “Have you experienced falls within the past 12 months?” Falls were defined as “an unexpected event in which the person comes to rest on the ground, floor or lower level” [18]. Participants who experienced at least one fall in the past 12 months were classified as fallers, and those with no falls in the past 12 months were classified as nonfallers.

#### 2.2.3. Physical Function

Walking speed was measured using a stopwatch on a flat and straight 10 m path at a comfortable walking speed. Two markers were used to indicate the start and end of the 10 m walk path, with a 2 m section to be traversed before passing the start marker, such that participants were walking at a comfortable pace at the first marker. Participants were also instructed to continue walking past the end of the 10-m path for a further two meters to ensure that the walking pace was kept constant throughout the task. Slow walking speed was defined as a walking speed < 1.0 m/s [10].

Muscle strength was assessed by dominant handgrip strength and measured using a Smedley-type handheld dynamometer (GRIP-D; Takei, Niigata, Japan). Muscle weakness was determined based on the Asian Working Group for Sarcopenia (AWGS) criteria for sarcopenia; handgrip strength was < 26.0 kg for men and < 18.0 kg for women [19].

Appendicular skeletal muscle mass was assessed using a multifrequency bioelectrical impedance analyser (BIA) (InBody 430, InBody Japan, Tokyo, Japan). The BIA instrument uses a tetrapolar, eight-point tactile electrode system that separately measures impedance of the arms, trunk and legs at three different frequencies (5, 50 and 250 kHz) for each segment [20]. The surface of the hand electrode was placed in contact with each of the five fingers, while the participant’s heels and forefoot were placed on the circular-shaped foot electrode. Participants held out their arms and separated their legs so that they did not contact other body parts during the assessment. Appendicular skeletal muscle mass was derived as the sum of the muscle mass of the four limbs, and the appendicular skeletal muscle mass index (ASMI; kg/m^2^) was calculated. Low skeletal muscle mass was determined based on the AWGS criteria for sarcopenia; ASMI < 7.0 kg/m^2^ for men and < 5.7 kg/m^2^ for women [19].

#### 2.2.4. Cognitive Function and Emotional Status

Cognitive assessments were conducted using the National Center for Geriatrics and Gerontology-Functional Assessment Tool (NCGG-FAT) to identify participants with MCI [21]. The NCGG-FAT comprises four domains: memory (immediate and delayed word list memory), visual motor speed (trail-making test-part A (TMT-A)), executive function (trail making test-part B (TMT-B)) and processing speed (symbol digit substitution test (SDST)). The NCGG-FAT has been shown to have high test-retest reliability [21], moderate-to-high criterion validity [21] and predictive validity for dementia [22], in community-dwelling older adults. Participants were given approximately 20 minutes to complete the tests. MCI was defined as a score below 1.5 SD of the age and education-specific means in one or more of the cognitive tests, based on the population sample of community-dwelling older adults [23]. Depressive symptoms was assessed using the 15-item geriatric depression scale (GDS-15) [24].

### 2.3. Statistical Analysis

Chi-square tests for cross-tabulation tables and student t-tests were used to examine differences in categorical and continuously scored measures, respectively, between the fallers and nonfallers, and the pain and nonpain groups. A multivariable logistic regression analysis with slow walking speed, muscle weakness, low skeletal muscle mass, MCI and GDS-15 as the independent variables and previous falls as the dependent variable were separately conducted in the pain and nonpain groups. Model 1 was unadjusted, and Model 2 was adjusted for relevant covariates: age, sex, education and medication use. All analyses were performed using SPSS 25.0 for Windows (SPSS Inc., Armonk, NY, USA), and significance levels were set at 0.05.

## 3. Results

### 3.1. Comparisons of Physical, Cognitive and Emotional Factors Between the Fallers and NonFallers Stratified by the Pain Status

Of the 789 participants, 421 (53.4%) reported pain, while 368 (46.6%) did not. The mean age of participants with and without pain was 75.8 and 73.7 years, respectively. Seventy-six participants in the pain group (18.1%) and 35 participants in the nonpain group (9.5%) were classified as fallers and nonfallers.

The pain group was significantly older than the nonpain group (*p* < 0.001), and the pain group comprised more women than men (*p* < 0.001) (Table 1). Compared with the nonpain group, the pain group took more prescribed medications (*p* < 0.001), had reduced handgrip strength (*p* = 0.008) and slower walking speed (*p* < 0.001) and had a greater proportion of slow walkers (*p* < 0.001). Regarding cognitive function and emotional status, the pain groups performed worse than the nonpain group in the immediate and delayed word list memory and SDST tests (*p* = 0.021, *p* = 0.006 and *p* = 0.014, respectively) and had higher GDS-15 scores (*p* < 0.001).

In the pain group, the fallers had significantly slower walking speed, lower SDST scores, a higher prevalence of MCI and higher GDS-15 scores than the nonfallers (*p* < 0.05) (Table 1). In the nonpain group, the fallers had a significantly higher prevalence of muscle weakness and lower skeletal muscle mass than the nonfallers (Table 1). No significant differences in the remaining variables were observed between the fallers and nonfallers in either pain group (*p* > 0.05).

### 3.2. Multivariable Logistic Regression to Determine the Fall Risks Of Physical, Cognitive and Emotional Factors in the Pain and NonPain Groups

In the pain group, MCI and GDS-15 scores were significantly associated with previous falls in both the unadjusted (Model 1) and adjusted models that included relevant covariates (Model 2) (Table 2). No physical function measures were significantly associated with previous falls in either model. In the nonpain group, muscle weakness and low skeletal muscle mass were significantly associated with previous falls in both the unadjusted (Model 1) and the adjusted models that included relevant covariates (Model 2). Slow walking speed, MCI and GDS-15 scores were not significantly associated with previous falls in either model.

## 4. Discussion

This study revealed older people with pain were significantly older and had slower gait speed, reduced muscle strength, impaired cognition and more depressive symptoms than those without pain. Further, the subgroup analysis unmasked some important information in that falls in the participants with pain were associated with cognitive and emotional factors, whereas falls in those without pain were associated with physical factors, including muscle weakness and low skeletal muscle mass in multivariable models adjusting for age, sex, education and medication use.

Although slow walking speed was identified as a risk factor for falls in the participants with pain in univariate analysis, this measure was not independently associated with falls in multivariable modelling. In contrast, the logistic regression analysis revealed the presence of MCI (assessed with tests of visual motor speed, memory, executive function and processing speed) and was independently associated with falls. Pain can interfere with attention in older adults [12,25], and insufficient or divided attention when negotiating environment hazards may lead to trips and slips [26,27]. Furthermore, pain is associated with slow processing speed [28], which is identified as a risk factor for falls in older adults [29]. Thus, it is possible that pain requires an attentional demand limiting the attentional resources allocated for avoiding daily life hazards and decision making, resulting in an increased risk of falls. Additionally, we found that depressive symptoms were associated with falls in those with pain. Depressive symptomatology has consistently been reported to increase the risk of falling in older people [8], and several studies have reported people with depression are more likely to develop chronic pain [14,30]. Further, pain-related fear can lead to avoidance behaviors and hypervigilance to bodily sensations followed by disability, disuse and depression [31], all factors that can exacerbate fall risk.

In the older adults without pain, those who reported falls were weaker and had lower skeletal muscle mass than those who did not report falls, and these two measures were independently associated with falls in the multivariate models. These findings are consistent with many previous studies conducted in older community-dwelling people [9,32], as well as complementary studies that have found that reduced muscle strength is associated with reduced balance control, slow sit-to-stand times and slow gait speed [33,34]. 

Our findings have implications for clinical practice. First, our findings that the pain group had slower gait speed and reduced muscle strength, compared to the nonpain group, provides insight into why this group is at increased fall risk [6,7]. Targeted exercise interventions could address these risk factors in addition to treatments for pain. Further, our subgroup analysis findings show that falls in participants with pain were associated with cognitive and emotional factors, including MCI and depressive symptoms, whereas falls in those without pain were associated with physical factors, including muscle weakness and low skeletal muscle mass. These findings suggest fall prevention interventions for older adults with and without pain may require special tailoring to address cognitive and emotional risk factors and physical factors for falls, respectively.

We acknowledge certain study limitations. First, our pain assessments were not detailed and additional assessments of pain intensity, duration and interference in activities of daily living may have provided further understanding of pain-related factors affecting falls. However, considering the high prevalence of chronic pain (25–76%) in community-dwelling older adults [35], the majority of our pain group participants would likely fall into this category. Second, the cross-sectional design precludes the prospective delineation of the relationship between falls and physical, cognitive and emotional factors among older adults with and without pain. Thus, our current study cannot confirm the causality between pain and risk of falling. Future research on prospective fall follow-up, and on detailed pain and physical, neuropsychological and functional assessments, is required to confirm the current findings. Furthermore, the retrospective recording of falls may have underestimated their true prevalence, although the overall proportion of fallers (16.4%) is consistent with most previous studies of fall incidence in older people undertaken in Japan.

## 5. Conclusions

Community-dwelling older people with pain were more likely to have depressive symptoms and impaired gait, strength and cognition than their peers without pain. Further, falls in those with pain were associated with cognitive and emotional factors whereas falls in those without pain were associated with physical factors (i.e., weakness and low skeletal muscle mass). Thus, fall-prevention interventions for older adults with pain may require tailoring to address cognitive and emotional risk factors for falls. Future research with prospective fall follow-up and detailed pain and physical, neuropsychological and functional assessments are required to confirm the current findings.

## Figures and Tables

**Table 1:** Comparisons of physical, cognitive and emotional factors between the fallers and nonfallers in the pain and nonpain groups.

Characteristics	Pain Group (*n* = 421)			Nonpain Group (*n* = 368)	
	Fallers (*n* = 76)	Nonfallers (*n* = 345)		Fallers (*n* = 35)	Nonfallers (*n* = 333)
Age (years)	77.1 (7.0)	75.5 (6.4)	^††^	74.1 (5.5)	73.6 (5.9)
Female, n (%)	53 (69.7)	235 (68.1)	^††^	23 (65.7)	195 (58.6)
Education (years)	11.0 (2.4)	11.0 (2.2)	^††^	10.9 (2.2)	11.4 (2.3)
Medications (n/day)	4.9 (4.5)	4.5 (4.5)	^††^	4.3 (7.1)	3.1 (4.6)
Physical function					
Slow walking speed, n (%)	18 (24.0)	60 (17.4)	^††^	3 (8.6)	25 (7.5)
Walking speed (m/s)	1.15 (0.25) **	1.24 (0.25)	^††^	1.29 (0.24)	1.33 (0.22)
Muscle weakness, n (%)	23 (30.3)	82 (24.5)		13 (37.1) **	59 (18.2)
Handgrip strength (kg)	23.1 (7.3)	23.5 (8.6)	^††^	23.0 (7.9)	25.2 (8.1)
Low skeletal muscle mass, n (%)	30 (40.0)	112 (33.7)		20 (57.1) **	120 (36.6)
ASMI (kg/m^2^)	6.2 (1.0)	6.3 (1.0)		6.1 (0.9)	6.4 (1.0)
Cognitive function					
MCI, n (%)	34 (44.7) **	95 (27.7)		12 (34.3)	96 (28.9)
Memory					
Immediate word list memory (score)	7.0 (1.6)	7.3 (1.6)	^†^	7.6 (1.2)	7.5 (1.4)
Delayed word list memory (score)	3.6 (2.0)	4.0 (2.1)	^††^	4.4 (2.1)	4.3 (2.1)
Visual motor speed					
TMT-A (s)	25.8 (8.7)	24.4 (10.8)		22.9 (6.3)	23.8 (15.0)
Executive function					
TMT-B (s)	70.0 (49.6)	54.7 (46.8)		57.4 (53.1)	51.2 (40.0)
Processing speed					
SDST (score)	36.0 (11.1) *	39.2 (11.3)	^†^	39.1 (9.4)	40.8 (11.9)
Emotional status					
GDS-15 (points)	3.7 (3.2) **	2.7 (2.5)	^††^	2.3 (2.6)	2.0 (2.1)

Data are presented as mean (SD) or number (percentage). ** *p* < 0.01, * *p* < 0.05 versus nonfallers, ^††^ *p* < 0.01, ^†^ *p* < 0.05 versus nonpain group; ASMI: appendicular skeletal muscle mass index. MCI: mild cognitive impairment. TMT: trail-making test. SDST: symbol digit substitution test. GDS-15: 15-item geriatric depression scale.

**Table 2:** Multivariable logistic regression analyses of the relationships between falls and physical, cognitive and emotional factors in the pain and nonpain groups.

Independent Variables	Dependent Variables: Previous Falls					
	Pain Group (n = 421)			NonPain Group (n = 368)		
	OR	95% CI	*p*-Value	OR	95% CI	*p*-Value
Slow walking speed						
Model 1	1.50	0.82–2.72	0.188	1.16	0.33–4.04	0.821
Model 2	1.13	0.69–2.54	0.405	1.15	0.30–4.31	0.841
Muscle weakness						
Model 1	1.34	0.77–2.32	0.298	2.66	1.27–5.59	0.010
Model 2	1.16	0.63–2.12	0.637	2.57	1.12–5.91	0.026
Low skeletal muscle mass						
Model 1	1.31	0.78–2.19	0.305	2.31	1.14–4.68	0.020
Model 2	1.01	0.60–1.85	0.848	2.18	1.03–4.60	0.041
MCI						
Model 1	2.11	1.27–3.52	0.004	1.28	0.61–2.68	0.508
Model 2	2.00	1.08–3.38	0.010	1.24	0.57–2.73	0.591
GDS-15						
Model 1	1.13	1.04–1.24	0.004	1.07	0.92–1.24	0.393
Model 2	1.14	1.04–1.24	0.005	1.04	0.89–1.21	0.620

Model 1: Unadjusted model, Model 2: Adjusted for age, sex, education and number of prescribed medications. OR: odds ratio. CI: confidence interval. MCI: mild cognitive impairment. GDS-15: 15-item geriatric depression scale.
